# Obstructive pneumonia with bronchial foreign body: A case report

**DOI:** 10.1016/j.heliyon.2023.e21362

**Published:** 2023-10-21

**Authors:** Hitomi Tanaka, Takatoshi Anno, Haruka Takenouchi, Katsumasa Koyama, Hideaki Kaneto, Niro Okimoto, Koichi Tomoda

**Affiliations:** a Department of General Internal Medicine 1, Kawasaki Medical School, Okayama, 700-8505, Japan; b Department of Diabetes, Endocrinology and Metabolism, Kawasaki Medical School, Kurashiki, 701-0192, Japan

**Keywords:** Obstructive pneumonia, Bronchial foreign body, Fish bone, Long-term cough, Past medical history

## Abstract

The age of predilection for foreign body aspiration into the lower airway shows a bimodal distribution, with the majority of cases occurring in children or infants and in the elderly. Although several pediatric airway foreign bodies have been summarized, in adults, bronchial foreign bodies are relatively uncommon. There are a variety of symptoms induced by airway foreign bodies, although the typical symptoms of some bronchial foreign bodies are cough. Bronchial foreign bodies, especially in the elderly, may have few symptoms and it is necessary for careful identification. Therefore, it is very important to carefully perform medical consultations about current and past medical history. Herein, we report a case of an elderly Japanese with obstructive pneumonia with a bronchial foreign body of fish bone with a long history of cough. It is known that people in some countries such as Japan have a habit of eating fish. Therefore, it is necessary to more carefully explore the possibility of some bronchial foreign body such as a fish bone, when we observe symptoms of persistent cough in such countries.

## 1 Introduction

Foreign body aspiration into the lower airway has been summarized in children [[1], [2], [3], [4]]. Bronchial foreign bodies are relatively uncommon in adults [5]. Various kinds of bronchial foreign bodies depend on countries and regions, age, and race. Many cases of endobronchial foreign bodies have been reported in infants, and such cases were caused by accidental aspiration. In general, retrieved objects include seeds, nuts, small toys, coins, pins in children, and bone fragments, medical instrument fragments, drug and dental appliances in adults [4].

There are a variety of symptoms induced by airway foreign bodies, although the typical symptoms of some bronchial foreign bodies are cough, wheezing, chest pain, hemoptysis, and high fever [6,7]. However, in some cases, there are no symptoms at all. Therefore, it is very important to carefully perform medical consultation about the current and past medical history such as persistent cough.

### 1.1 Case

**Fig. 1:**
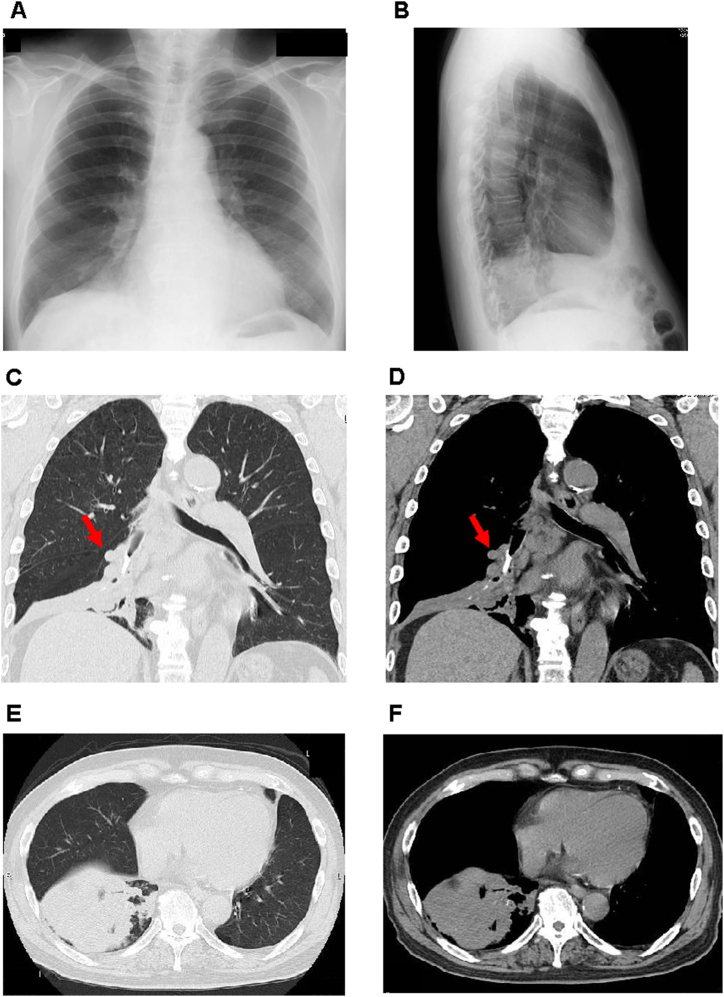
Findings in chest radiograph (A and B) and chest computed tomography (CT) (C–F). Chest radiograph showed an increase of opacity in the right lower lung. Chest CT revealed obstructive pneumonia in the right lower lung and bronchial foreign body (red arrow). (For interpretation of the references to color in this figure legend, the reader is referred to the Web version of this article.)

An 80-year-old Japanese man was referred to our hospital with symptoms of a 7-month history of cough and pneumonia. He visited his private physician at first due to a persistent cough and was treated with antibiotics at that time. His symptoms of cough had not improved but he had no symptoms except for such persistent cough. His symptom of cough was sometimes accompanied by white viscous sputum without bleeding, but most of them were dry coughs. Once his symptoms of cough became paroxysmal without some trigger, he had a persistent cough. However, once the cough improved, he did not have any symptoms at all. Therefore, he hesitated to continue the treatment. Since he suddenly had neck pain a few weeks before which was unrelated to coughing, a chest radiograph was taken by his private physician. His chest radiograph showed a slight increase in opacity in the right lower lung at that time (Fig. 1A and B), although an airway foreign body was not clearly observed. His medical history was myocardial infarction at the age of 68 and percutaneous coronary intervention with stenting was placed. He was a smoker (pack-years = 2 packs/day × 48 years) before experiencing myocardial infarction at the age of 68, but after then he stopped smoking. He had no remarkable family history. He lived with his wife without any neurological challenges. On admission, his vital signs were as follows: temperature, 35.9 °C; blood pressure, 126/64 mmHg; heart rate, 70 beats/min; oxygen saturation, 97 % (room air). His laboratory data were as follows: white blood cell count, 8590/μL (neutrophil, 70.5 %); red blood cell count, 455 × 10^4^/μL; hemoglobin, 14.1 g/dL; platelet, 18.4/μL. Liver and renal function was almost within the normal range as follows: aspartate aminotransferase (AST), 18 U/L; alanine transaminase (ALT), 13 U/L; alkaline phosphatase (ALP) 219 U/L; γ-glutamyl transpeptidase (γ-GTP), 13 U/L; lactate dehydrogenase (LDH), 176 U/L; creatinine (CRE), 1.12 mg/dL; blood urea nitrogen (BUN), 20 mg/dL. Lung-associated data and tumor markers were as follows: cytokeratin 19 fragment (CYFRA), 1.7 ng/mL; pro-gastrin-releasing peptide (ProGRP), 49.9 pg/mL; QuantiFERON, negative; mycobacterium avium complex antibody, negative; carcinoembryonic antigen (CEA), 2.0 ng/mL; carbohydrate antigen 19-9 (CA19-9), 17.7 U/mL; prostate specific antigen (PSA), 1.401 ng/mL. We performed chest computed tomography (CT) to get more information. As shown in Fig. 1C-F, chest CT revealed obstructive pneumonia surrounding area was edematous in the right lower lung and a bronchial foreign body which was consistent with high density.

**Fig. 2:**
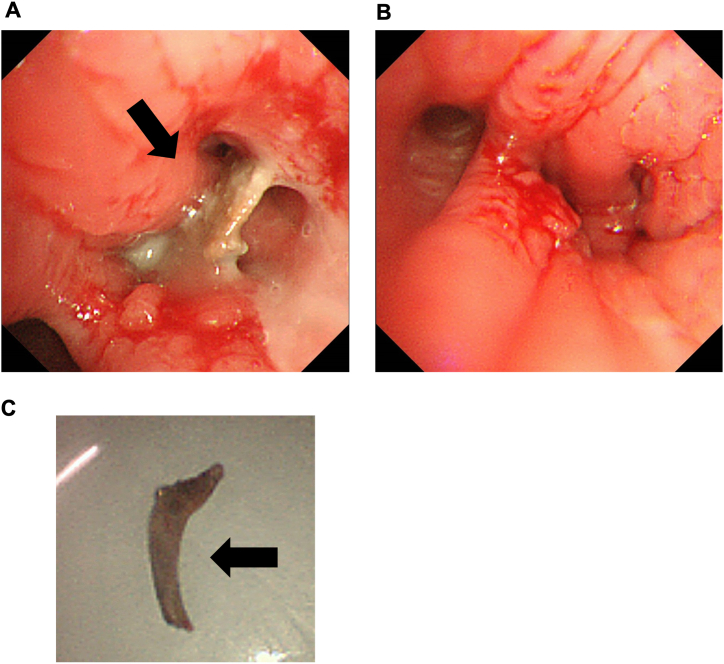
Findings in bronchoscopy. In bronchoscopy, a fish bone was detected as an intrabronchial foreign body in the right lower lung. A; An intrabronchial fish bone (black arrow). B; Intrabronchi after removal of a fish bone. C; A fish bone.

Therefore, we performed more careful medical consultations about the current and past medical history once more. The patient clearly remembered that he had mis-swallowing approximately 11 months before because he had choked on eating yellowtail fish. However, he felt this episode was unrelated and he hesitated to propose this thing because he visited his private physician for the first time after four months. Therefore, we considered that his intrabronchial calcified structure on chest CT could be a fish bone. The bronchial foreign body may have been present for more than 10 months, and in such cases, granulation and edema around the bronchial foreign body may occur. We performed flexible bronchoscopy using the fibreoptic technique under local anesthesia and detected obstruction of the basal segmental bronchus's lumen by a fish bone as an intrabronchial foreign body and edematous formation in the right lower lung and finally removed it from the bronchi (Fig. 2A-C). A small amount of white-colored sputum was aspirated from the right basal bronchus and peripheral bronchus until the location of the bronchial foreign body. Approximately 10 days later, a follow-up bronchoscopy was performed, which revealed no abnormalities.

## 2 Discussion

Herein, we report a case of obstructive pneumonia with a bronchial foreign body of fish bone with an 11-month history of bronchial foreign body and a 7-month history of cough. There are several reports of obstructive pneumonia due to some bronchial foreign bodies. It has been noted that there are several differences in the features of foreign body cases in children between Asians and Westerners, suggesting that ethnic food habits may influence the type of foreign bodies [8,9]. The patients with pharyngeal and esophageal foreign bodies come from every age, and in particular, elderly patients are often involved in esophageal foreign bodies because of mis-swallowing. In general, airway foreign bodies are observed in children under 10 years of age and the second peak is observed in patients over age 70, and fish bones are the most common in Japanese children [10,11], although some Japanese cases in elderly patients were reported in the Japanese language. Similarly, it was reported that foreign bodies in the respiratory tract were caused by fish fin. Therefore, it seems that food habits are very important for foreign bodies in Asia [12]. In addition, although, many cases of the foreign body were infants and the elderly worldwide [6], laryngeal and tracheal foreign bodies account for 2–11 % of all foreign bodies, and more peripheral foreign bodies under subglottic such as bronchial foreign bodies are about 10 % of total laryngeal and tracheal foreign bodies. Moreover, 93 % of such cases result in respiratory failure [13]. In this case, the bronchial foreign body, but not pharyngeal and esophageal foreign bodies, is rare even in elderly patients, and the bronchial foreign body of fish bone is characteristic in Asia. In addition, it is rare that this case had obstructive pneumonia for 7 months only with a history of cough.

The typical symptoms of some bronchial foreign bodies are cough, wheezing, chest pain, hemoptysis, and high fever [6,7], but in some cases, there are no symptoms at all. For this reason, in older adults, symptoms are often attributed to medical conditions, especially when no clear history of aspiration is apparent, and can be longstanding [14]. It should be noted that bronchial foreign bodies are generally more likely to occur in adults when they are more elderly age, the use of sedative or hypnotic medications, or presence of some medical condition [15,16]. The clinical information about the bronchial foreign body is limited. Adult bronchial foreign body is easily misdiagnosed and delayed in diagnosis, especially for older patients, because of the non-specificity of its clinical feature and usually more obscure [7]. In addition, it is sometimes difficult to detect such a foreign body on a chest radiograph [17,18], although some imaging tests are usually effective [19]. In the present case, once his symptoms of cough became paroxysmal without some trigger, he had a persistent cough. However, once the cough improved, he did not have any symptoms at all. Therefore, he hesitated to continue to have the treatment. This is one of the main reasons why this patient was not properly treated. Lin et al. reported clinical features of bronchial foreign bodies in elderly patients [20] and recommended flexible bronchoscopy as the first-line approach to similar patients, especially those with aspiration history and unexplained pneumonia. In our case as well, bronchoscopy was useful for the diagnosis and treatment of obstructive pneumonia with bronchial foreign body of the fish bone.

This case report clearly indicates that it is very important to carefully perform medical consultations about the current and past medical history, when there is a persistent cough of unknown cause. In addition, this case is a good opportunity to remind the first-touch physician. Taken together, we should bear in mind the possibility of obstructive pneumonia due to some bronchial foreign body in such a situation where a subject choked on eating even when there is a long-time episode. In addition, people in some countries and regions such as Japan have a habit of eating fish. It is necessary to more carefully consider the possibility of some bronchial foreign body such as a fish bone, when we observe symptoms of persistent cough, especially if the cause is unknown.

## Funding

This research received no specific grant from any funding agency in the public, commercial, or not-for-profit sectors.

## Data availability statement

The authors confirm that the data supporting the findings of this study are available within the article [and/or] its supplementary materials.

## Ethics statement

The study was conducted according to the guidelines of the Declaration of Helsinki. The reporting of this study conforms to CARE guidelines [21].

## Written informed consent

Written informed consent was obtained from the patient for publication of this case report and accompanying images. A copy of the written consent is available for review by the Editor-in-Chief of this journal on request.

## CRediT authorship contribution statement

**Hitomi Tanaka:** Conceptualization, Data curation, Formal analysis, Investigation, Writing – original draft. **Takatoshi Anno:** Conceptualization, Data curation, Formal analysis, Investigation, Methodology, Writing – original draft, Writing – review & editing. **Haruka Takenouchi:** Conceptualization, Data curation, Formal analysis, Investigation. **Katsumasa Koyama:** Conceptualization, Data curation, Formal analysis, Investigation. **Hideaki Kaneto:** Writing – original draft, Writing – review & editing. **Niro Okimoto:** Writing – review & editing. **Koichi Tomoda:** Writing – review & editing.

## Declaration of competing interest

The authors declare that they have no known competing financial interests or personal relationships that could have appeared to influence the work reported in this paper.
